# Effect of Cu_2_P_2_O_7_ on the Formation of Black Micro-Arc Oxidation Coating on AZ31 Magnesium Alloy

**DOI:** 10.3390/ma19040811

**Published:** 2026-02-20

**Authors:** Jian Chen, Hongtao Li, Bo Chen, Kun Wang

**Affiliations:** College of Materials Science and Engineering, Nanjing Tech University, Nanjing 211816, China; chenjian243654@163.com (J.C.);

**Keywords:** micro-arc oxidation, Cu_2_P_2_O_7_, black coating, CuO

## Abstract

**Highlights:**

**What are the main findings?**

- Adding 4–5 g per liter of Cu_2_P_2_O_7_ can change the coating color from grayish white to uniform black.
- Copper is incorporated into the coating in the form of copper oxide (CuO), as confirmed by XPS/EDS, which results in the coating presenting a black color.
- The phase composition consists of MgO, MgSiO_3_, and Mg; however, the content of MgO decreases with increasing Cu_2_P_2_O_7_ concentration.
- The optimal dosage of 4 g/L enhances the coating density by thickening the dense layer and reducing the pore size.

**What are the implications of the main findings?**

- The one-step preparation of a black micro-arc oxidation coating has been achieved, simplifying the surface treatment process of magnesium alloys.
- Clarifying the coloring mechanism of Cu_2_P_2_O_7_ is conducive to the development of functional colored surfaces.
- Even if there are trade-offs in terms of corrosion, this approach can still support applications in the aerospace/automotive fields and offer aesthetically pleasing solutions.

**Abstract:**

Magnesium alloys require protective surface coatings for widespread application, with micro-arc oxidation (MAO) being a prominent technique. However, conventional MAO coatings are typically gray or light-colored, necessitating secondary treatments for specific colors like black, which complicates the process. This study aims to develop a one-step method for fabricating black MAO coatings on AZ31 magnesium alloy by introducing cupric pyrophosphate (Cu_2_P_2_O_7_) as a colorant into a silicate-based electrolyte. As the Cu_2_P_2_O_7_ concentration increased from 0 to 5 g/L, the coating color transitioned from grayish-white to pink, then brownish-black, achieving a uniform black appearance at 4–5 g/L. XPS and EDS analyses confirmed the incorporation of copper as CuO, identified as the primary coloring agent. XRD indicated that the phase composition remained MgO, MgSiO_3_, and Mg, although the MgO content decreased. Microstructural analysis showed that an optimal concentration of 4 g/L enhanced coating compactness by thickening the dense layer and reducing pore size. However, electrochemical tests revealed that the incorporation of CuO significantly increased the corrosion current density, thereby reducing the coating’s corrosion resistance compared to the unmodified coating. This work successfully demonstrates the one-step fabrication of black MAO coatings, elucidates the coloration mechanism involving CuO formation, and provides insights into the trade-off between aesthetic functionalization and corrosion performance.

## 1. Introduction

Magnesium alloys, as one of the lightest metal structural materials [1,2], demonstrate great application potential in aerospace, the automotive industry, 3C products, and biomedical fields due to their excellent specific strength, specific stiffness, good biocompatibility, and recyclability [3,4,5,6]. However, the chemical properties of magnesium alloys are highly reactive, and their relatively negative standard electrode potential makes them prone to corrosion in conventional service environments, severely restricting the full utilization of their performance and further promotion of their application. Therefore, developing efficient and reliable surface protection technologies for magnesium alloys is the key to realizing their engineering applications.

Micro-arc oxidation (MAO) technology, as an electrochemical surface treatment technology for in situ growth of ceramic oxide films on valve metals, can obtain ceramic coatings with adjustable composition, structure, and properties on magnesium alloys, effectively enhancing their corrosion resistance, wear resistance, and insulation [7,8,9,10,11]. However, traditional micro-arc oxidation coatings usually present as gray or light gray, and in scenarios requiring specific appearances such as black, secondary coloring treatment is often necessary, which not only increases the complexity and cost of the process but may also affect the integrity and adhesion of the coating. Therefore, developing a technology that can directly prepare a specific black micro-arc oxidation coating on magnesium alloys in one step has significant theoretical significance and practical value [12].

The color of MAO coatings is typically achieved by introducing specific elements into the electrolyte, which form colored oxides or phases within the coating. For example, introducing titanium (via K_2_TiF_6_) results in black TiO_2_ and Mg_2_TiO_4_ [13], while iron (from K_3_[Fe(C_2_O_4_)_3_]) produces black Fe_2_O_3_/Fe_3_O_4_ [14]. Similarly, adding carbon nanoparticles directly can form a black coating on aluminum [15].

Among various colorants, copper salts have particular potential because copper oxide (CuO) exhibits a unique black color, which has been confirmed in the research by Wu et al. using copper sulfate [16]. The general principle of introducing copper ions during polymer electroplating has been recognized [17]. However, a thorough review reveals significant knowledge gaps in the use of copper acid phosphate (Cu_2_P_2_O_7_). Its stability in alkaline silicate electrolytes, the precise form of copper ions under metal-assisted oxidation conditions, and the impact on the coating formation mechanism and final performance are still poorly understood. Additionally, there is a lack of systematic research on the correlation between the concentration of copper acid phosphate (Cu_2_P_2_O_7_) and the evolution of coating structure and functional performance and the chemical state and distribution of the incorporated copper.

Therefore, to address these unresolved issues, this study specifically conducted an in-depth investigation of this new coloring agent, Cu_2_P_2_O_7_. This study aimed to achieve a uniform black MAO coating on AZ31 alloy in a one-step process, systematically elucidate the effects of Cu_2_P_2_O_7_ concentration on the coating color, microstructure, phase composition, and corrosion resistance, and fundamentally clarify the coloring mechanism by determining the chemical state and distribution of the incorporated copper.

## 2. Materials and Methods

### 2.1. Experimental Preparation

The samples used in this experiment were 40 mm × 40 mm × 4 mm rectangular AZ31 magnesium alloy specimens. The element content of AZ31 die-cast magnesium alloy is shown in Table 1. Before the experiment, the surface of the samples was polished with SiC sandpaper to an average roughness of 0.1 μm, and then the samples were placed in anhydrous ethanol and ultrasonically cleaned to remove surface oils. Finally, the samples were washed with deionized water and dried before being prepared for use.

The electrolyte consists of 8 g/L NaSiO_3_, 10 g/L KF, 8 g/L NaOH, 5 g/L NaKC_4_H_4_O_6_, and Cu_2_P_2_O_7_ at concentrations of 0 g/L, 1 g/L, 2 g/L, 3 g/L, 4 g/L, and 5 g/L, respectively. The prepared coatings are named S0, S1, S2, S3, S4, and S5. The AZ31 magnesium alloy specimen is the anode, and the stainless steel container is the cathode. A pulse power supply (JCL-21KW, JCL, Chengdu, China) with a maximum power of 20 KW is used in the micro-arc oxidation process under a constant current mode. The constant current density is 2.0 A⋅dm^−2^, the duty cycle is 10%, and the frequency is 800 Hz. The treatment time is 10 min. During the MAO process, a magnetic stirring system is used to enhance the mass transfer in the electrolyte. A water cooling system is used to cool the reaction electrolyte to room temperature.

### 2.2. Coating Characterization

The physical phase composition of the micro-arc oxidation coating was analyzed by using the diffraction of incident X-rays (XRD, D/Max-2400 X-ray diffractometry, Rigaku, Tokyo, Japan). A copper target (Kα1 = 0.15406 nm) was selected as the anode target. The scanning rate was 10°/min, the grazing angle was set at 4°, the scanning range was set from 10 to 90°, and the step size was set to 0.02°. X-ray photoelectron spectroscopy (K-Alpha, Thermo Fisher Scientific, Waltham, MA, USA) was carried out to analyze the chemical constituents and states. The scanning electron microscope (JSM-7900F, JEOL, Tokyo, Japan) was used to observe the surface morphology of the micro-arc oxidation-treated samples, and the elemental composition of the coating was analyzed and characterized using energy dispersive spectroscopy (JSM-IT500A, JEOL, Tokyo, Japan). The coating thickness was measured using a handheld eddy current thickness gauge (FMP20, Helmut Fischer GMBH, Sindelfingen, Germany). The blackness of the micro-arc oxidation coating was measured using an LS171 colorimeter (LS171, Shenzhen Linshang Technology, Shenzhen, China). The corrosion resistance of the micro-arc oxidation coating was tested using an electrochemical workstation (PGSTAT302 N, Metrohm Autolab, Utrecht, The Netherlands). To ensure the reliability of the results, all micro-arc oxidation experiments and subsequent characteristic tests were repeated three times. The reported color values represent the average values calculated from the three repeated tests. For the electrochemical tests, for each coating type, at least three potentiodynamic polarization measurements were conducted to ensure the repeatability of the results.

## 3. Results

### 3.1. The Influence of Cu_2_P_2_O_7_ on the Basic Characteristics of the Electrolyte

**Figure 1:**
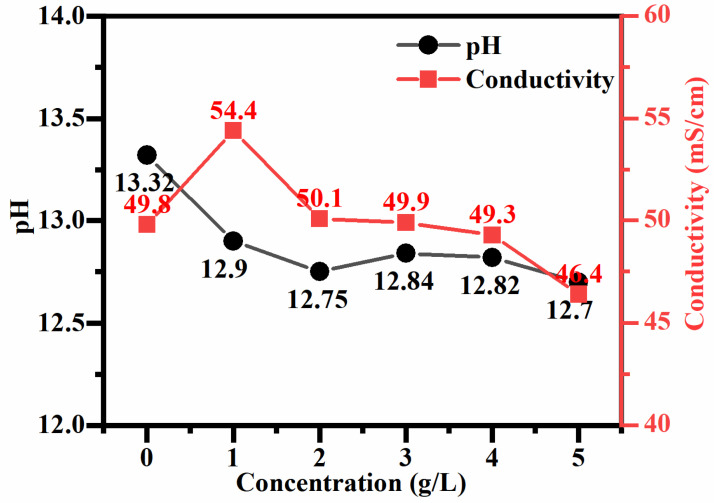
pH and conductivity of the silicate electrolyte after adding different concentrations of Cu_2_P_2_O_7_.

The electrolyte used for PEO processing is a silicon-based electrolyte with different concentrations of cupric pyrophosphate added. The introduction of cupric pyrophosphate may cause changes in the electrolyte environment. The conductivity and pH after adding different concentrations of cupric pyrophosphate are shown in Figure 1. It can be clearly seen that the pH of the solution shows an overall downward trend, which is due to the reaction of cupric pyrophosphate with hydroxide ions to form copper hydroxide precipitation, and then copper hydroxide forms a soluble copper complex with potassium tartrate sodium. The reactions are shown in Equations (1) and (2). Due to the consumption of hydroxide ions, the pH of the solution decreases. Initially, the increase in conductivity was attributed to the dissociation and complexation reactions of Cu_2_P_2_O_7_, which introduced additional free ions (sodium ions, potassium ions, phosphate ions). At higher concentrations, the subsequent decrease occurred mainly due to the consumption of free hydroxide ions, which have high conductivity. Additionally, these ions may form larger, less mobile ion complexes or colloidal substances, thereby reducing the overall mobility of ions. The changes in pH value and conductivity are not merely incidental phenomena but are the key factors determining the mechanism of copper element integration. At the optimal concentration (approximately 4 g per liter), sufficient tartaric acid ligands can keep copper in an insoluble [Cu(C_4_H_4_O_6_)_2_]^2−^ complex form. These negatively charged complexes migrate towards the anode under the influence of an electric field and uniformly participate in plasma electrochemical reactions, thereby forming finly dispersed CuO within the dense coating. On the contrary, at excessive concentrations (5 g per liter), insufficient ligands will lead to the precipitation of Cu(OH)_2_. These solid particles randomly move to the coating surface, causing local intense discharge and damaging the integrity of the coating.

(1) $${Cu}_{2}P_{2}O_{7}+6NaOH\to 2Cu{\left(OH\right)}_{2}+2{Na}_{3}PO_{4}+H_{2}O,$$

(2) $$Cu{\left(OH\right)}_{2}+2KNaC_{4}H_{4}O_{6}=K_{2}\left[Cu\left(C_{4}\right.{\left.H_{4}O_{6}\right)}_{2}\right]+2NaOH+2H_{2}O,$$

### 3.2. The Influence of Cu_2_P_2_O_7_ on the Apparent Color of the Black Coating Formed by Micro-Arc Oxidation on Magnesium Alloys

**Figure 2:**
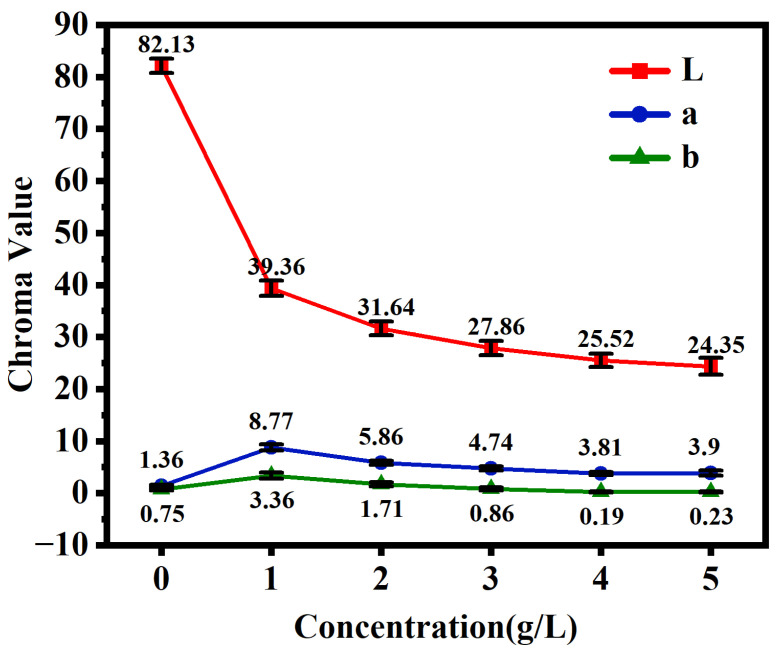
The Lab values of micro-arc oxidation coatings formed by different concentrations of Cu_2_P_2_O_7_.

Figure 2 presents the Lab color values of micro-arc oxidation coatings formed at varying concentrations of Cu_2_P_2_O_7_ in the silicate-based electrolyte. Analysis of the Lab data reveals that the L* value progressively decreases with increasing Cu_2_P_2_O_7_ concentration, indicating a continuous enhancement in coating blackness. These findings demonstrate that the incorporation of copper ions into the silicate electrolyte significantly influences the coloring of micro-arc oxidation coatings on AZ31 magnesium alloy.

**Figure 3:**
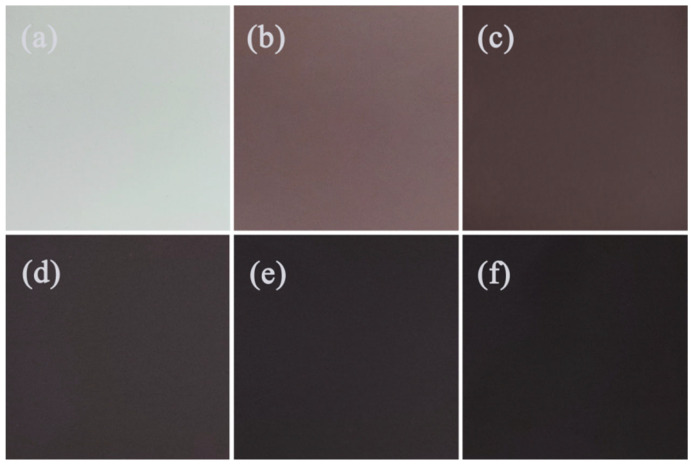
Macroscopic surface appearance of micro-arc oxidation coatings fabricated with varying concentrations of Cu_2_P_2_O_7_: (**a**) S0, (**b**) S1, (**c**) S2, (**d**) S3, (**e**) S4, (**f**) S5.

Figure 3 presents the macroscopic surface morphology of micro-arc oxidation coatings formed in silicate-based electrolytes with varying concentrations of Cu_2_P_2_O_7_. The coating grown in the absence of Cu_2_P_2_O_7_ exhibits a white appearance (Figure 3a), whereas the color of the MAO coating on AZ31 magnesium alloy progressively darkens with increasing Cu_2_P_2_O_7_ concentration. At a concentration of 1 g/L, a pink-colored coating is obtained (Figure 3b). When the concentration is increased to 2–3 g/L, the coating turns brownish-black (Figure 3c,d). In the research on black functional coatings, an L* value below 30 is usually regarded as the benchmark value for a black surface [18]. In this article, the L values of different black coatings are all approximately 29, which is much higher than those of the current study. Upon further increasing the Cu_2_P_2_O_7_ concentration to 4–5 g/L, a uniformly black MAO coating is successfully fabricated on the AZ31 alloy surface (Figure 3e,f). These visual observations are in good agreement with the Lab color value analysis presented in Figure 2. The results clearly demonstrate that copper ions participate in complex plasma-electrochemical reactions during the MAO process, ultimately leading to the formation of a black ceramic coating on the AZ31 magnesium alloy surface.

### 3.3. The Influence of Cu_2_P_2_O_7_ on the Phase Composition and Microstructure of the Black Coating of Magnesium Alloys

**Figure 4:**
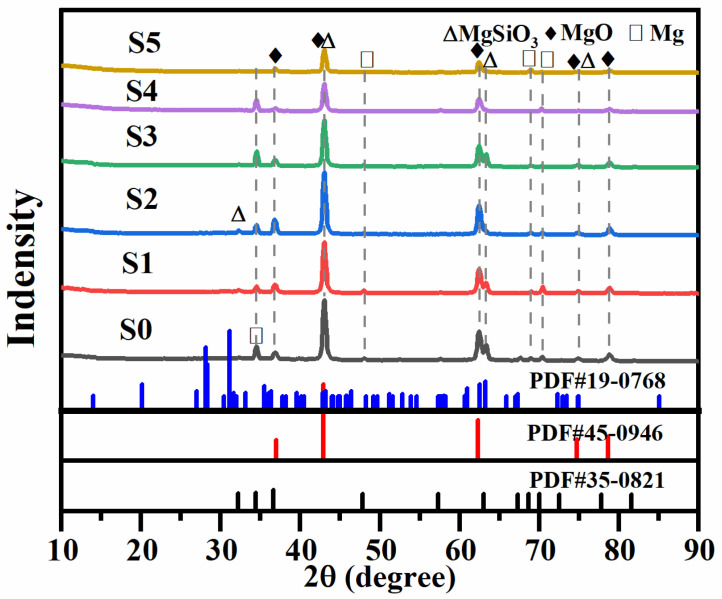
XRD patterns of micro-arc oxidation coatings fabricated using silicate electrolytes with varying concentrations of Cu_2_P_2_O_7_.

Figure 4 shows the XRD diffraction patterns of micro-arc oxidation coatings grown in silicate electrolytes with different concentrations of Cu_2_P_2_O_7_. It can be seen from the figure that the micro-arc oxidation coatings are mainly composed of Mg (PDF#35-0821), MgSiO_3_ (PDF#19-0768) and MgO (PDF#45-0946). The increase in the concentration of Cu_2_P_2_O_7_ does not change the phase composition of the coating, which remains MgSiO_3_, MgO and Mg. However, the peak intensity of MgO gradually decreases, indicating that the relative content of MgO in the coating gradually decreases. The CuO phase was not detected in the copper-containing coatings; this phenomenon may be due to the fact that the copper oxide is amorphous, or that its content may be below the detection limit.

**Figure 5:**
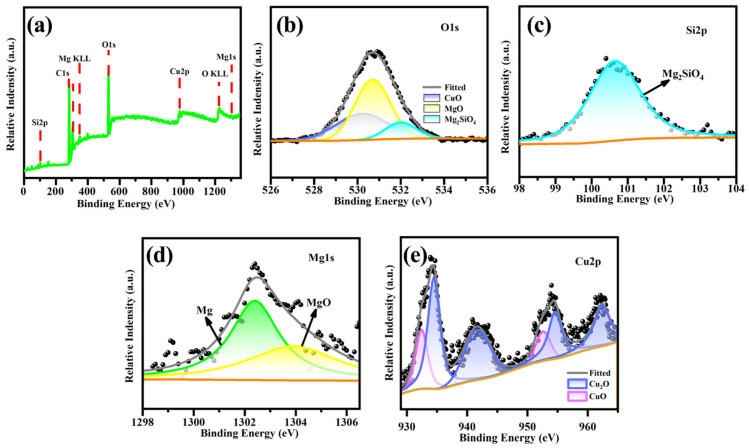
XPS spectra of micro-arc oxidation coatings fabricated in a silicate-based electrolyte containing 5 g/L Cu_2_P_2_O_7_: (**a**) XPS spectra, (**b**) O1s, (**c**) Si2p, (**d**) Mg1s, (**e**) Cu2p.

To further confirm the existence state of Cu in the micro-arc oxidation coating, XPS analysis was conducted on the micro-arc oxidation coating formed in 5 g/L Cu_2_P_2_O_7_ solution, and the corresponding results are shown in Figure 5. The O 1 s spectrum was divided into three peaks at 532.0 eV, 530.7 eV and 530.2 eV (Figure 5b), corresponding to Mg_2_SiO_4_, MgO and CuO, respectively [19,20,21]. A peak appeared at 100.6 eV in the Si 2p spectrum (Figure 5c), which existed in the form of Mg_2_SiO_4_. The binding energies of the two peaks in the Mg 1 s spectrum were 1302.45 eV and 103.8 eV, corresponding to Mg and MgO, respectively. As shown in Figure 5, the XPS spectra of Cu 2p have characteristic peaks of CuO at 932.9 eV and 952.6 eV and those of Cu_2_O at 934.5 eV and 954.8 eV, which is consistent with the literature reports [22]. Based on the analysis of XPS and EDS results, it can be concluded that the formation of the black micro-arc oxidation coating on magnesium alloy is due to the participation of Cu in the Cu_2_P_2_O_7_ in the reaction to generate black CuO.

**Figure 6:**
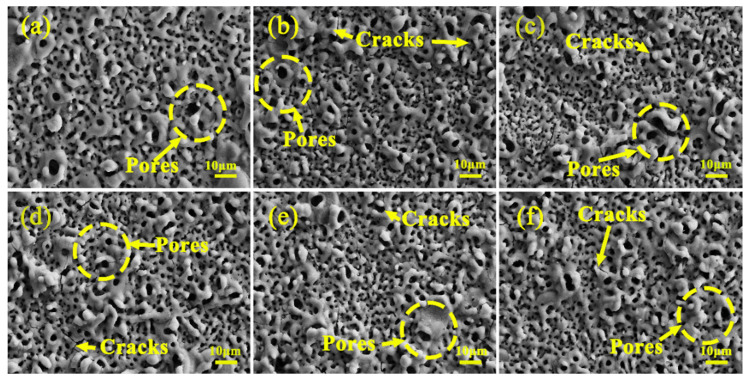
Surface morphologies of micro-arc oxidation coatings fabricated in silicate electrolytes with varying concentrations of Cu_2_P_2_O_7_: (**a**) S0, (**b**) S1, (**c**) S2, (**d**) S3, (**e**) S4, (**f**) S5.

Figure 6 presents the surface morphology of micro-arc oxidation coatings fabricated in silicate electrolytes containing varying concentrations of Cu_2_P_2_O_7_. The results reveal that all coatings exhibit a characteristic porous structure, with pore size markedly influenced by the electrolyte composition, which is consistent with findings reported in the literature [23,24]. As observed in the images, the coating surfaces are densely populated with micro-pores and micro-cracks. These features originate from the high-temperature and high-pressure plasma discharge microenvironments, where magnesium ions released from the alloy substrate react with ionized oxygen species in the electrolyte to form metal oxides. Rapid solidification of the outer oxide layer upon contact with the cool electrolyte, coupled with residual thermal stresses in the inner layers, leads to the formation of micro-cracks. Additionally, oxygen gas generated during water electrolysis becomes entrapped within the growing coating; the resulting pressure differential between the interior and exterior promotes bubble expansion and rupture, expelling molten oxide and gas to form surface micro-pores. Evidence of localized melting around certain pores further supports intense plasma discharging during coating growth. In the absence of Cu_2_P_2_O_7_, most surface pores range from 2 to 3 μm in diameter, with a few exceeding 5 μm. With increasing Cu_2_P_2_O_7_ concentration, the average pore size decreases progressively, reaching a minimum at 4 g/L, accompanied by partial pore filling. However, the number of pores and cracks increases concurrently. Notably, when the concentration reaches 5 g/L, incomplete complexation leads to the formation of blue Cu(OH)_2_ precipitates in the electrolyte, compromising its stability. Under the influence of the electric field and electrophoretic forces, these precipitates migrate toward the anode surface, inducing localized tip discharge and concentrating discharge energy, which ultimately results in the formation of larger, irregular pores, as clearly illustrated in Figure 6f.

Table 2 presents the EDS results of micro-arc oxidation coatings fabricated in silicate electrolytes with varying concentrations of Cu_2_P_2_O_7_. White-spot EDS analysis reveals that the coatings are primarily composed of Mg, O, and Si elements. The Mg originates from the magnesium alloy substrate, whereas O and Si are derived from the silicate electrolyte. In comparison to the unmodified coating (S0), the surface Cu content increases progressively with increasing Cu_2_P_2_O_7_ concentration, indicating a direct dependence on the concentration of free Cu^2+^ complex ions available in the electrolyte. Oxygen not only reacts with Mg to form MgO but also participates in the formation of CuO through reaction with Cu species. CuO exhibits good electrical conductivity and is widely applied in catalytic and ceramic materials. The characteristic black appearance of the micro-arc oxidation coating is attributed to the uniform incorporation and distribution of Cu, likely in the form of CuO, throughout the coating matrix.

**Figure 7:**
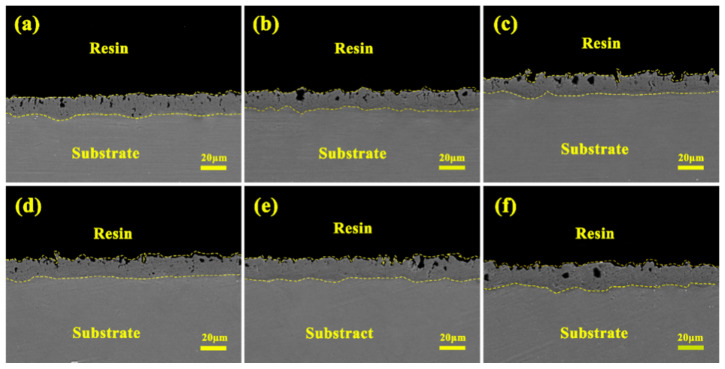
Cross-sectional morphologies of micro-arc oxidation coatings fabricated in a silicate-based electrolyte with varying concentrations of Cu_2_P_2_O_7_: (**a**) S0, (**b**) S1, (**c**) S2, (**d**) S3, (**e**) S4, (**f**) S5.

Corresponding to the porous structure revealed by the surface morphology (Figure 6), Figure 7 presents the cross-sectional morphologies of micro-arc oxidation coatings fabricated in silicate electrolytes with varying concentrations of Cu_2_P_2_O_7_. The results demonstrate that the coatings typically consist of an inner dense layer and an outer porous layer [25,26,27]. Without the addition of Cu_2_P_2_O_7_, the coating has the thinnest dense layer and the smoothest cross-section (Figure 7a). As the concentration of Cu_2_P_2_O_7_ increases to 4 g/L, the thickness of the inner dense layer significantly and continuously increases (Figure 7b–e), which is attributed to the accelerated migration of complexed Cu^2+^ under the enhanced electric field and its effective electrochemical deposition in the discharge channel. Meanwhile, the number of pores in the outer porous layer gradually decreases, and the structure tends to be denser. However, when the concentration reaches 5 g/L, the Cu(OH)_2_ precipitation caused by the instability of the electrolyte triggers local intense discharge, which disrupts the uniformity and continuity of the dense layer and leads to the formation of macroscopic defects (Figure 7f). Therefore, the cross-sectional analysis indicates that an appropriate amount of Cu_2_P_2_O_7_ (≤4 g/L) optimizes the coating structure by promoting the densification of the inner layer, while excessive addition has the opposite effect.

**Figure 8:**
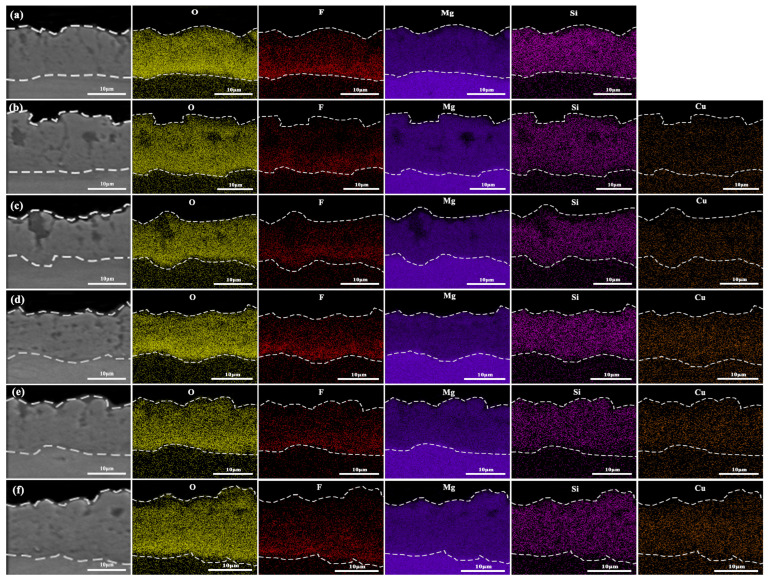
Cross-sectional EDS elemental mapping of micro-arc oxidation coatings fabricated in a silicate-based electrolyte with varying concentrations of Cu_2_P_2_O_7_: (**a**) S0, (**b**) S1, (**c**) S2, (**d**) S3, (**e**) S4, (**f**) S5.

Figure 8 presents the EDS elemental distribution of micro-arc oxidation coatings fabricated in silicate electrolytes with varying concentrations of Cu_2_P_2_O_7_. As shown in the figure, the white coating is primarily composed of Mg, O, Si, and F elements, originating from the magnesium alloy substrate and the silicate-based electrolyte. Upon the addition of Cu_2_P_2_O_7_, Cu is detected in the black micro-arc oxidation coatings, indicating that the resulting coating consists of Mg, O, Si, F, and Cu. Specifically, Mg originates from the substrate, Si is supplied by Na_2_SiO_3_ in the electrolyte, F is derived from KF, O is predominantly generated through water electrolysis, and Cu is introduced via Cu_2_P_2_O_7_. The presence of Cu within the coating confirms its participation in the oxidation reactions during micro-arc oxidation, leading to the formation of a black-colored coating likely containing CuO. Elemental mapping reveals that O, Mg, and Si are uniformly distributed throughout the coating, suggesting their active involvement in the oxide growth process. In contrast, the F element exhibits a gradient distribution, decreasing gradually from the inner to the outer layer. This indicates that F^−^ ions are preferentially adsorbed by Mg in the substrate and readily migrate into the coating interior through discharge channels, which is consistent with the findings reported by Dong et al. [28] Compared with the unmodified coating (S0), the Cu content increases progressively with increasing Cu_2_P_2_O_7_ concentration. This trend arises because higher concentrations provide more Cu^2+^ complex ions in solution, enhancing their participation in plasma electrolytic oxidation and concurrent coloring processes, thereby strengthening Cu distribution across the coating cross-section.

### 3.4. Effects of Cu_2_P_2_O_7_ on the Corrosion Resistance of Black Micro-Arc Oxidation Coatings on Magnesium Alloys

**Figure 9:**
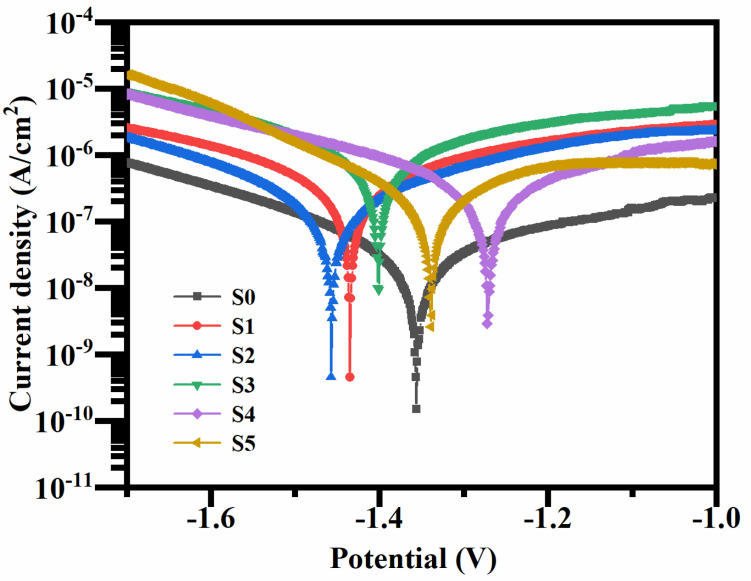
Potentiodynamic polarization curves of micro-arc oxidation coatings fabricated with varying concentrations of Cu_2_P_2_O_7_ in a silicate-based electrolyte.

The electrochemical polarization curve of the MAO coating formed in the silicate-based electrolyte containing Cu_2_P_2_O_7_ is shown in Figure 9. Generally speaking, the corrosion potential reflects the stability of the coating, while the corrosion current density corresponds to its corrosion rate. A higher corrosion potential and a lower corrosion current density indicate better corrosion resistance. Compared with the coating prepared under the condition without Cu_2_P_2_O_7_, the coating prepared under the condition with Cu_2_P_2_O_7_ has a higher corrosion current density, indicating a decrease in corrosion resistance. This decline in corrosion performance can be attributed to two interrelated factors. Firstly, the micro-arc oxidation process itself generates a coating with a porous and cracked morphology, which itself weakens its barrier function. Secondly, more importantly, the introduction of a highly conductive CuO phase introduces a large number of cathode sites in the coating matrix. In the reference coating (S0), although it is porous, the oxide phase (MgO, MgSiO_3_) is insulating, limiting the electrochemical activity. In contrast, in the copper-containing coating, the infiltrated electrolyte can quickly connect the CuO sites on the cathode with the Mg matrix on the anode, forming an efficient electrochemical cell. Therefore, the synergistic effect between the porous microstructure and the electrochemically active CuO phase leads to a significant reduction in corrosion resistance.

As shown in Table 3, although the corrosion current density of this coating is about one to two orders of magnitude higher than that of the copper-free coating, it still forms an important protective barrier compared to the bare substrate. According to the classic corrosion engineering standards (Fontana, 1986) [29], a corrosion rate lower than 0.025 mm/year (corresponding to a corrosion current density of approximately 1.1 × 10^−6^ A/cm^2^) is considered “excellent” grade and is suitable for indoor and mild environments. In this study, even the worst corrosion current density of the copper-containing coating is still lower than this threshold. In a controlled indoor environment or a mild pressure environment, for the sake of aesthetics and practicality, it is a feasible solution because its unique black appearance is the primary requirement. Additionally, if one wants to improve its corrosion resistance, sealing treatment is also a good option.

### 3.5. Analysis of the Film Formation Mechanism of Black Micro-Arc Oxidation Coatings

**Figure 10:**
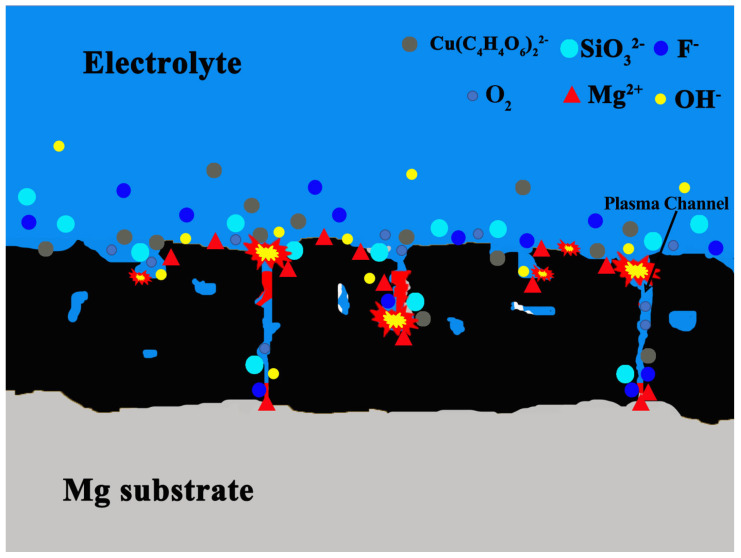
Schematic illustration of the formation mechanism of black micro-arc oxidation coatings in a Cu_2_P_2_O_7_-based electrolyte system.

The formation mechanism of the black micro-arc oxidation coating using Cu_2_P_2_O_7_ as a coloring agent is illustrated in Figure 10. In the absence of sodium potassium tartrate, Cu_2_P_2_O_7_ in the alkaline silicate electrolyte readily reacts with sodium hydroxide to form Cu(OH)_2_ precipitates, whereas the free [Cu(C_4_H_4_O_6_)_2_]^2−^ complex ions are more favorable for the formation of black copper oxides during the micro-arc oxidation process. The chelated Cu^2+^ ions migrate into the coating interior through discharge channels under the influence of the electric field and undergo electrochemical reactions to form the CuO phase, which is corroborated by XPS analysis. Since micro-arc oxidation preferentially initiates breakdown and discharging at locally weak regions of the coating, prolonged oxidation time leads to progressive closure of internal discharge channels, promoting the growth of the dense inner layer. As the oxidation voltage increases continuously, intensified micro-arc activity enhances surface micro-pore enlargement and micro-crack propagation, ultimately resulting in the development of an outer porous layer.

## 4. Conclusions

1. The study successfully achieved controllable color modulation of the coating. In the absence of Cu_2_P_2_O_7_, the coating appeared grayish white; as the Cu_2_P_2_O_7_ concentration increased, the coating color gradually evolved from pink to brownish black. A uniformly black coating was obtained at a concentration of 4–5 g/L, accompanied by a significant reduction in its L* value (lightness), indicating enhanced darkening.
2. XPS analysis confirmed that the copper species from the electrolyte participated in chemical reactions during the high-temperature and high-pressure micro-arc discharge process and were successfully incorporated into the coating as black CuO. This incorporation is the primary cause of the coating’s black appearance. EDS results demonstrated that the Cu content in the coating increased steadily with rising Cu_2_P_2_O_7_ concentration in the electrolyte and exhibited a uniform spatial distribution.
3. Although adding approximately 4 g per liter of Cu_2_P_2_O_7_ helps to form a denser coating and smaller surface pores, simultaneously incorporating CuO phases into the coating matrix will significantly reduce its corrosion resistance due to the galvanic effect. Therefore, the refinement of the microstructure is not sufficient to compensate for the adverse electrochemical effects introduced by the colorant.
4. Despite structural improvements, the corrosion resistance of all coatings containing Cu_2_P_2_O_7_ was systematically inferior to that of the reference coating without additive. Potentiodynamic polarization curves revealed that the corrosion current density of the copper-containing coatings increased by one to two orders of magnitude. This degradation is primarily attributed to the formation of the CuO phase within the coating, which exhibits a more positive electrochemical potential and high electrical conductivity. Upon electrolyte infiltration, this conductive phase establishes an efficient galvanic couple with the active magnesium substrate, thereby significantly accelerating the anodic dissolution of magnesium.

## Figures and Tables

**Table 1:** Elements contained in AZ31 alloy and their contents (wt.%).

Element	Al	Zn	Si	Mn	Fe	Cu	Mg
**Contents**	3.12	1.04	0.006	0.44	0.001	0.001	bal.

Note: Data tested by the authors.

**Table 2:** Surface element contents of Cu_2_P_2_O_7_ coatings with different concentrations.

Sample	Content of Elements (wt.%)			
	Mg	O	Si	Cu
S0	45.31	45.78	8.92	0
S1	47.40	44.58	7.08	0.93
S2	47.62	43.79	7.23	1.36
S3	47.39	43.18	7.56	1.87
S4	46.57	42.79	7.83	2.81
S5	45.56	42.91	8.05	3.48

**Table 3:** Polarization curve parameters of micro-arc oxidation coatings.

Sample	Ecorr (mV vs. Ag/AgCl)	icorr (A/cm^2^)
S0	−1357 ± 15	(8.03 ± 0.38) × 10^−9^
S1	−1433 ± 20	(6.01 ± 0.29) × 10^−8^
S2	−1449 ± 21	(6.93 ± 0.39) × 10^−8^
S3	−1396 ± 18	(1.21 ± 0.45) × 10^−7^
S4	−1276 ± 11	(1.41 ± 0.32) × 10^−7^
S5	−1349 ± 22	(2.24 ± 0.33) × 10^−7^

## Data Availability

The original contributions presented in this study are included in the article. Further inquiries can be directed to the corresponding author.
